# Reply to Paranhos-Baccalà et al. Comment on “Cuesta et al. An Assessment of a New Rapid Multiplex PCR Assay for the Diagnosis of Meningoencephalitis. *Diagnostics* 2024, *14*, 802”

**DOI:** 10.3390/diagnostics14171885

**Published:** 2024-08-28

**Authors:** Genoveva Cuesta, Pedro Puerta-Alcalde, Andrea Vergara, Enric Roses, Jordi Bosch, Climent Casals-Pascual, Alex Soriano, Maria Ángeles Marcos, Sergi Sanz, Jordi Vila

**Affiliations:** 1 Department of Clinical Microbiology, Hospital Clinic, 08036 Barcelona, Spain; gcuesta@clinic.cat (G.C.); vergara@clinic.cat (A.V.); rosas@clinic.cat (E.R.); jobosch@clinic.cat (J.B.); ccasals@clinic.cat (C.C.-P.); mamarcos@clinic.cat (M.Á.M.); 2 Department of Infectious Diseases, Hospital Clínic-IDIBAPS, University of Barcelona, 08007 Barcelona, Spain; puerta@clinic.cat (P.P.-A.); asoriano@clinic.cat (A.S.); 3 Department of Basic Clinical Practice, Faculty of Medicine, University of Barcelona, 08007 Barcelona, Spain; sergi.sanz@isglobal.org; 4 CIBER Enfermedades Infecciosas (CIBERINFEC), Instituto de Salud Carlos III, 28029 Madrid, Spain; 5 Institute of Global Health of Barcelona (ISGlobal), 08036 Barcelona, Spain; 6 CIBER Epidemiología y Salud Pública (CIBERESP), Instituto de Salud Carlos III, 28029 Madrid, Spain

We appreciate the interest and reflections of Paranhos-Baccalà and colleagues in our recent article published in *Diagnostics* [1]. They are correct regarding the overall sensitivity of the Biofire^R^ Filmarray^R^ ME panel, as other studies have generally demonstrated high sensitivity for most of the microorganisms included in the panel [2]. However, there is some controversy specifically concerning HSV-1, as while some studies have shown good sensitivity, others have not. In a meta-analysis by Trujillo et al. [3], they alluded to the fact that the Biofire^R^ Filmarray^R^ ME panel showed a suboptimal sensitivity for HSV-1, emphasizing that it is very limited for ruling out these viruses. In our study, the sensitivity of the Biofire^R^ ME panel was 85%; however, the specificity was 57%, associated with a group of samples which gave positive results with the Biofire^R^ Filmarray^R^ ME panel, whereas they were negative when using our in-house PCR for HSV and the QIAstat-Dx ME panel. Tansarli et al. [4], in a meta-analysis, reported a high proportion of false negative HSV-1 diagnoses but also seven false positive HSV-1 diagnoses among 150 positives. Furthermore, in a multicenter evaluation of Biofire^R^ Filmarray^R^ by Leber et al. [5], which involved 1560 prospectively collected CSF specimens, positive HSV-1 results were found. Of these, two were considered true positives, while the other two were considered false positive, as the latter samples were from patients without final diagnoses of HSV encephalitis.

The authors also suggested that the high percentage of immunosuppressed patients might have increased the risk of false positive results for HSV-1. However, we are unsure about the reason for this potential association, and we have checked that, at least in our paper, the rates of Biofire^R^ Filmarray^R^ results were not different between immunosuppressed and non-immunosuppressed patients. We believe that the overall high rates of false positive results were mainly due to only mildly altered CSF fluids being analyzed, potentially decreasing the positive pre-test likelihood. However, the QIAstat-Dx ME panel seemed to be less affected by this fact. They also mentioned that concomitant HSV-1 detection in cases of other documented causes of infection might be due to the re-activation of HSV-1 [6]. Four out of the seven patients in our study who tested positive for HSV-1 with the Biofire^R^ Filmarray^R^ ME panel and negative with the QIAstat-Dx ME panel did not take acyclovir. Three of them recovered, and the death of the fourth was attributed to cardiogenic shock with myocardial involvement in a patient with severe hepatic necrosis and Wernicke’s encephalopathy. In our study, the clinical evaluation (by the physicians in charge) of some patients concluded that it was not meningoencephalitis and indeed, the patients did not receive acyclovir. Of course, we cannot completely exclude the reactivation of an HSV as a consequence of other stress or infection, but even in this circumstance, no treatment would be necessary. In the future, it would be of interest to determine the presence of HSV DNA in spinal fluid from patients with other well-documented conditions requiring an analysis of spinal fluid (e.g., subarachnoid hemorrhage).

Paranhos-Baccalà et al. referred to the fact that the high percentage of positive samples studied is not representative of typical clinical laboratory conditions and may skew the results. We agree that this may be a limitation of the study, and this was stated in the limitations paragraph; however, we do not see why this would skew our results. In our paper, we admit that this study has several limitations, as mentioned, although we do not think that any of them would directly affect our results. In fact, the only limitation that could affect the results, but in a contrary way, would have been that the samples were frozen; however, that would have generated false negative results likely associated with the lysis of the virus and the degradation of the genome. They also mentioned that it could have been good to rerun the samples again through the BioFire platform; however, the samples that showed false positive results obtained using the Filmarray ME panel were not reanalyzed using the same technique due to the volume limitation of the CSF samples, with a preference for using another technique to confirm the results. 

We celebrate that the kinetics of amplification can now be visualized through the Biofire platform. However, when we mentioned that it is important to check the amplification curve, this was not only to visualize the Ct but also to see if the curve had the appropriate shape. Paranhos-Baccalà et al. also outline the fact that many of our samples had polymicrobial detections, which is very intriguing. It should be highlighted that all polymicrobial detection was observed using the Biofire^R^ Filmarray^R^ ME panel and that, indeed, after analyzing the sample through the QIAstat-Dx ME panel or conventional methods, only one microorganism was found to be the cause of the encephalitis.

The authors mentioned that “clinical correlation remains essential; diagnosis should not rely solely on laboratory results”. We could not agree more with this statement, and we think that the strength of our paper is mainly based on the final clinical diagnosis that showed that the patients with HSV-1-positive samples with the Biofire ME panel and negative samples with QIAStat did not have herpetic encephalitis. In addition, all six samples with HSV-1 positivity and the four samples with HSV-2 positivity for both platforms did indeed show herpetic encephalitis. However, we acknowledge that our cohort includes a relatively small number of samples, and thus larger comparative studies are needed to confirm our findings.
